# Unusual Presentation of Castleman’s Disease Encroaching on the Brachial Plexus

**DOI:** 10.7759/cureus.16981

**Published:** 2021-08-07

**Authors:** Rahil H Shah, Maureen Cioffi-Lavina, Abraham Jaguan, Marcia Varella

**Affiliations:** 1 Internal Medicine, Jackson Memorial Hospital/University of Miami, Miami, USA; 2 Department of Pathology, Mercy Hospital, Miami, USA; 3 Department of Otolaryngology, Mercy Hospital, Miami, USA; 4 Department of Medical and Population Health Sciences Research, Florida International University Herbert Wertheim College of Medicine, Miami, USA

**Keywords:** castleman disease, brachial plexus, supraclavicular mass, angiofollicular lymphoid hyperplasia

## Abstract

Castleman’s disease is an uncommon benign lymphoproliferative disorder that commonly involves the mediastinum. We report an unusual case that involves the presentation of unicentric Castleman’s disease in a 52-year-old female. The patient had a supraclavicular mass extending onto the brachial plexus. The approach to the treatment and plan for supraclavicular masses is complex due in part to the extensive list of differential diagnoses possible. In this case specifically, while the mass was ultimately determined to be benign, post-surgery, the location of the mass intraoperatively made for a very technically challenging and complex dissection. Post surgical resection, the patient reported no complications. This case highlights the importance of clinical judgement, imaging and surgical technique in removing a mass encroaching on the brachial plexus.

## Introduction

Castleman’s disease (CD), also known as angiofollicular lymphoid hyperplasia, is a rare, benign lymphoproliferative disorder first described by Dr. Benjamin Castleman in 1954 [1]. CD is classified as unicentric Castleman’s disease (UCD) or multicentric Castleman’s disease (MCD) depending on the extent of lymph node involvement. UCD, the more common presentation, then further subdivides onto a spectrum that ranges from plasma cell histopathologic to hyaline vascular histopathologic. The MCD type is subdivided into positive or negative for human herpes 8 virus [2].

Typically, CD presents as a mediastinal mass, however, there are reports of CD arising in suprarenal, mesentery, pancreas, neck, among various other locations [3-5]. In this case report, we present a unique presentation of unicentric CD as a supraclavicular mass that imposes on the brachial plexus causing neural symptoms in a patient. Due to its precarious positioning relative to the anatomy of the neck and shoulder, it proved to be a very technically challenging dissection.

## Case presentation

A 52-year-old female was referred to an Ear Nose and Throat (ENT) clinic with a progressively growing right lateral neck mass. The mass was noticed two years before the ENT visit date and described as slowly growing. Patient underwent a fine needle aspiration biopsy after noticing the mass, which was inconclusive. Since then, patient has noticed the neck mass progressively increasing in size. She noticed occasional paresthesia in her right arm. The patient denied weight loss, night sweats, fatigue or nausea. She also denied any muscle spasms, hoarseness, or neck stiffness. On physical exam, a 4 x 3 cm supraclavicular mass (level IV) rubbery, non-tender, non-erythematous, and with no associated warmth was noted on the right side. Otherwise, general examination was unremarkable.

The differential diagnosis for supraclavicular lymphadenopathy included lymphoma, malignancy, mycobacterial infection, Castleman’s disease, Kikuchi’s disease, and Kimura disease, among others. Imaging was ordered to visualize and position the mass.

**Figure 1:**
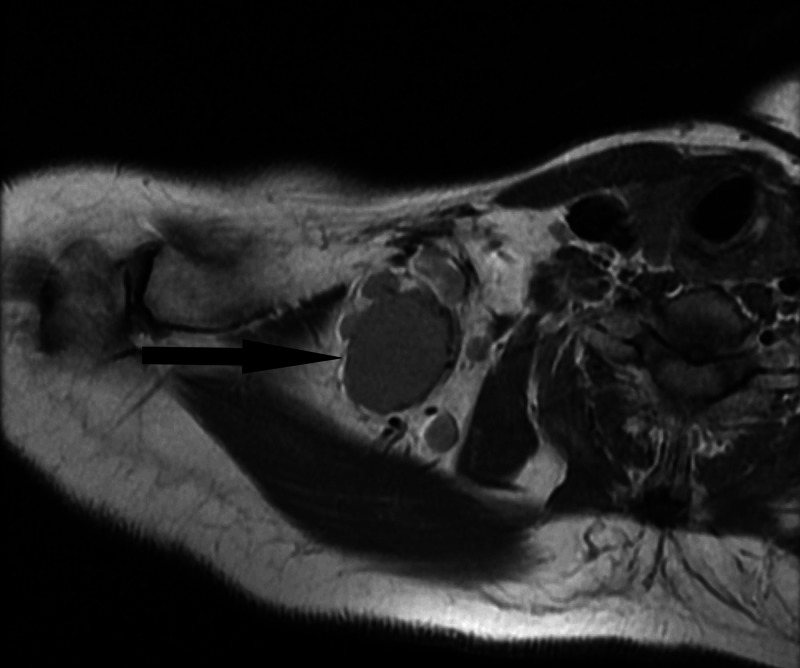
Right brachial plexus proton density-weighted MRI showing a 3.3cm by 2.6cm mass.

**Figure 2:**
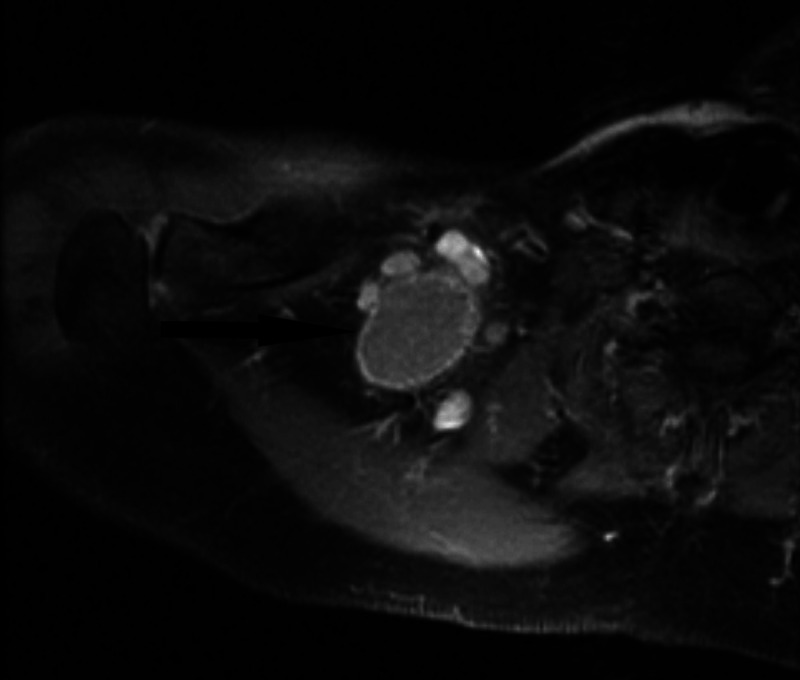
Right brachial plexus MRI T1 weighted showing the 3.3cm x 2.7cm mass.

CT scan showed a well-circumscribed neck mass that appeared solid and homogenous in nature. The mass was positioned very close to the brachial plexus. Malignancy was suspected and surgical removal was advised. Due to the precarious location of the mass, a follow-up MRI with contrast was ordered to better assess the relationship with the brachial plexus and surrounding structures (Figures 1, 2). The MRI was reviewed with a neuroradiologist and was confirmed close proximity of the mass to the brachial plexus. Ultimately, surgical removal was recommended and the risks and benefits were explained to the patient. The surgery was performed six weeks after the MRI results.

Over the six-week interval, the mass had grown in size and imposed further on the brachial plexus. After incision and dissection, the mass was visualized. The mass had grown deeper into the neck than previously anticipated, measuring at the time of surgery 4.5 x 3 x 2 cm and extending into the subclavicular region. The mass attached itself to the subclavian artery and vein, resulting in a very difficult dissection. The mass was subsequently finger dissected from the brachial plexus to avoid excessive stimulation of the underlying nerves. Finally, after carefully detaching it, the mass was removed from the supraclavicular region. The mass was ovoid-shaped, tan-brown in color, non-tender and surrounded by a thick fibrous capsule.

**Figure 3:**
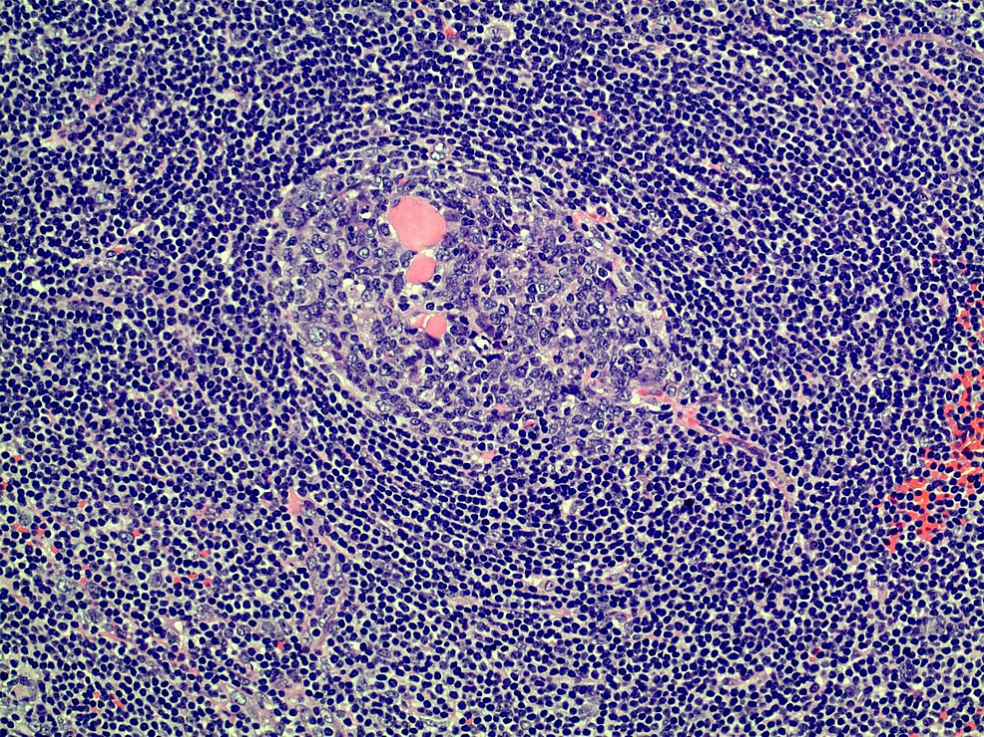
Histologic images for Catleman disease shows an atretic germinal center traversed by penetrating vessel – (lollipop follicle). Mantle zones are thickened with lymphocytes arranged in layers – (onion skin appearance) (hematoxylin-eosin, original magnification x40)

**Figure 4:**
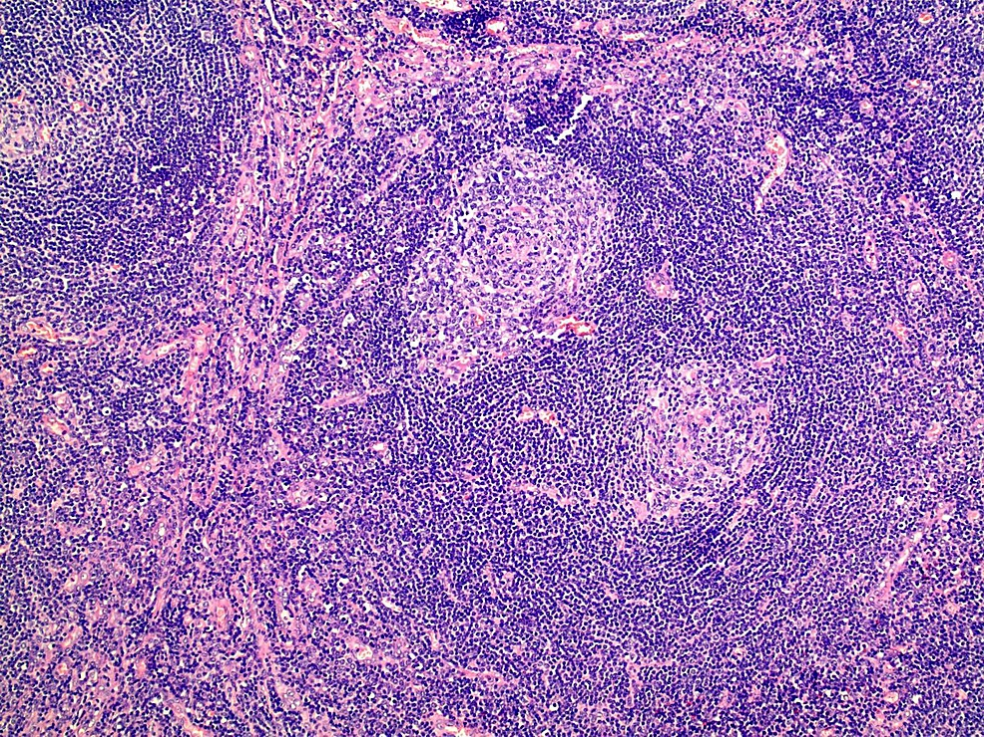
Histopathological imaging shows a mantle zone containing more than one germinal center (hematoxylin-eosin, original magnification x40)

Histopathology analysis showed an enlarged lymph node with prominent vascular proliferation. Numerous germinal centers were present, characterized by thickened mantle zones comprised by lymphocytes arranged in layers of “onion skin appearance” and atretic germinal centers traversed by vessels “lollipop follicles” (Figures 3, 4). Human herpesvirus-8 (HHV8) immunohistochemical stains were negative. These findings were consistent with unicentric Castleman’s disease, hyaline vascular variant. 

Patient presented at 14 days post-op with no complaints or concerns. She had no neurological symptoms or muscle weakness. She was feeling well and plans to continue regular follow-up for prevention of future advanced masses. Typical follow-up regimen for UCD includes annual PET/CT scans, and lab studies (complete blood count, lactate dehydrogenase, complete metabolic panel, interleukin-6, C-reactive protein, serum-free light chain assay, and quantitative immunoglobulins.) If disease free for five years, surveillance imaging may be discontinued [6].

## Discussion

Castleman’s disease (CD), also known as angiofollicular lymphoid hyperplasia, is a rare, benign lymphoproliferative disorder [1]. CD is divided into two main subtypes depending on the number of regions of lymph nodes involved: unicentric and multicentric. Unicentric Castleman’s disease (UCD) is characterized on a spectrum that ranges from plasma cell histopathology to hyaline vascular histopathology. Multicentric Castleman’s disease (MCD) is further divided into two groups depending on the presence of human herpesvirus-8: HHV8+ MCD and HHV8- MCD, also referred to as idiopathic MCD (iMCD). UCD occurs in 16/1,000,000 people. HHV8+ MCD occurs in every 5/1,000,000 people [2].

Of UCD cases, the hyaline vascular subtype comprises the majority at about 90% of cases [7]. It is histologically characterized by large lymphoid follicles, vasculature with prominent hyalinization, and circular layers of lymphocytes. The “onion skinning” of the rings of mantle zone lymphocytes and the single penetrating central vessel producing a “lollipop-like” appearance are characteristic features of the hyaline vascular form of Castleman's disease [8]. The plasma cell type is characterized by the substantial growth of polyclonal plasma cells in interfollicular areas. Afflicted patients can present with anemia, pyrexia, night sweats, and hyperglobulinemia [9].

HHV8+ MCD is the most clearly defined and understood variant of MCD. HHV8+ MCD is a waxing and waning febrile illness, commonly occurring in immunocompromised populations (e.g. HIV+), characterized by diffuse lymphadenopathy, splenomegaly, and anemia [6]. iMCD does not have a known pathogenesis, however, it is associated with high levels of IL-6. iMCD can present with two separate paraneoplastic syndromes. The first is characterized by polyradiculoneuropathy, organomegaly, endocrinopathy, monoclonal plasma cell neoplasm, and skin changes (POEMS). The second is characterized by thrombocytopenia, anasarca, fever, renal insufficiency, and organomegaly (TAFRO). POEMS has been seen in 25% [10] to 32% [11] of patients with MCD; whereas TAFRO is much rarer and was seen in one out of 79 patients (1.3%) in a Beijing study [10] and nine out of 43 patients (21%) in an MD Anderson study [12].

Treatment is unique to each subtype of CD. Since UCD is a localized mass, surgical resection is curative in those patients with the hyaline vascular type, whereas chemotherapy, radiation or steroid therapy is preferred in patients with the plasma cell type [9]. When considering treatment for HHV8+ MCD, the recommended treatment is rituximab, which shows a 90% survival rate. For iMCD, the recommendation is an IL-6 blocker such as tocilizumab or siltuximab.

There has only been one previous report of the relationship between CD and the brachial plexus [13]. They reported a mass growing onto the brachial plexus causing Parsonage-Turner syndrome. Parsonage-Turner syndrome (neuralgic amyotrophy) is an uncommon disorder of the peripheral nervous system characterized by the sudden onset of extreme pain in the upper extremity followed by rapid multifocal motor weakness and atrophy, and a slow recovery in months to years. It may develop after a viral illness, mild trauma, or surgery. The most common theory as to its cause is that of a viral-induced, immune-mediated process [14].

An analysis of previous cases of Castleman’s disease presenting in the head and neck demonstrated the risks of surgical removal [15]. Eleven cases of UCD were examined with various subtypes and presentations. Post resection, one patient had slight facial paralysis due to the involvement of the facial nerve. Another patient reported shoulder pain after removal. Another case demonstrated neural symptoms related to CD. They reported a unique case of trigeminal neuropathy in a patient with mesenteric UCD [16]. Once the mass was removed, the patient’s symptoms subsided with no recurrence.

Differentials for supraclavicular masses are very broad and often require sampling or removal to determine a definitive diagnosis. Castleman’s disease should be considered when diagnosing a lymphoid mass. Additionally, proper and thorough imaging should always be done prior to surgical removal when facing a potentially difficult dissection to prevent patient morbidity.

## Conclusions

We present an unusual case of Castleman's disease that extended onto the brachial plexus. Castleman's disease presents in many ways, ranging from nonspecific symptoms to potential paraneoplastic syndromes, but very rarely has it been seen involving the brachial plexus. Histopathological analysis is needed to determine the subtype of Castleman's disease which will determine treatment. This case highlights the importance of clinical judgement, imaging and surgical technique when faced with a supraclavicular mass.
